# Passive Gamma-Ray and Neutron Imaging Systems for National Security and Nuclear Non-Proliferation in Controlled and Uncontrolled Detection Areas: Review of Past and Current Status

**DOI:** 10.3390/s19112638

**Published:** 2019-06-11

**Authors:** Hajir Al Hamrashdi, Stephen D. Monk, David Cheneler

**Affiliations:** Department of Engineering, Lancaster University, Lancaster LA1 4YW, UK; s.monk@lancaster.ac.uk (S.D.M.); d.cheneler@lancaster.ac.uk (D.C.)

**Keywords:** passive radiation detection, gamma-ray, neutron, illicit trafficking, national security, non-proliferation

## Abstract

Global concern for the illicit transportation and trafficking of nuclear materials and other radioactive sources is on the rise, with efficient and rapid security and non-proliferation technologies in more demand than ever. Many factors contribute to this issue, including the increasing number of terrorist cells, gaps in security networks, politically unstable states across the globe and the black-market trading of radioactive sources to unknown parties. The use of passive gamma-ray and neutron detection and imaging technologies in security-sensitive areas and ports has had more impact than most other techniques in detecting and deterring illicit transportation and trafficking of illegal radioactive materials. This work reviews and critically evaluates these techniques as currently utilised within national security and non-proliferation applications and proposes likely avenues of development.

## 1. Introduction

Due to the hazardous ionising and activating nature of neutron and gamma radiation, there is a requirement to control and monitor the radiological materials, which produce them. Neutron and gamma-ray detection can directly lead to the identification of radiological sources in general, including nuclear materials. Due to the potential of these materials to be developed into nuclear weapons, these substances can pose direct threats to national security, and so are of great interest.

Illicit trafficking of nuclear materials and other radiological sources present a global threat that international organisations such as the IAEA (International Atomic Energy Agency) are forced to tackle frequently [1,2]. The IAEA Incident and trafficking database reported 3235 confirmed incidents of nuclear and other radioactive materials out of regulatory control between 1993 and 2017. Of these incidents, 278 were associated with trafficking or malicious use of materials such as highly-enriched uranium, plutonium and plutonium–beryllium neutron sources [2]. This issue highlights the importance of the effective control of nuclear and radiation materials at national and international cross points such as borders, ports and airports.

Effective application of radiation detection techniques requires knowledge of the environment in which the technology will be implemented, and the associated circumstances. In a controlled detection area such as an airport checkpoint, border line checkpoint, cargo inspection checkpoint or air cargo inspection, the space, and in most cases the physical contact time, allow for a reasonable level of flexibility. In an uncontrolled detection area such as buffer zones, airports terminals, train stations and public roads, space and physical contact time are less flexible and require more advanced detection technologies [3].

This review compares the various technologies utilised in radiation portal monitoring (RPM) of illicit radioactive materials including radiation sources, by-product materials and nuclear materials, with a view of identifying their advantages and limitations.

## 2. Radioactive Materials, Nuclear Materials and Radiation Sources:

Radioactive materials are defined by the IAEA as materials being designated in the national law or by a regulatory body as being subject to regulatory control because of their radioactivity [4]. Nuclear material is similarly defined as:

- Any plutonium isotope concentration except that with 80% or more of ^238^Pu,
- Uranium enriched in the isotopes ^233^U or ^235^U,
- Uranium containing the mixture of isotopes as occurring in nature other than in the form of ore or ore-residue,
- Any material containing one or more of the above [4].

**Figure 1:**
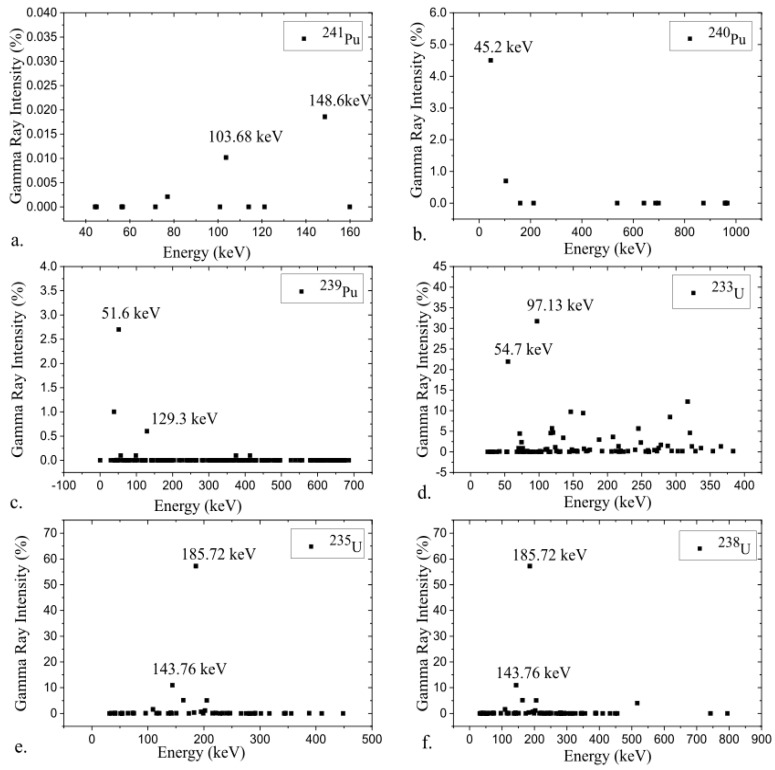
Characteristic gamma spectrum and gamma peaks of nuclear materials isotopes a. ^241^Pu, b. ^240^Pu, c. ^239^Pu, d. ^233^U, e. ^235^U, f.^238^U (Data source: Idaho National Engineering and Environmental Laboratory [9]).

A radiation source is usually defined as artificially refined radioactive material produced outside the nuclear fuel cycles of research and power reactors [4,5]. The choice of radiation detection technology employed is primarily based on the radiation type being emitted, the amount of radiation, the energy spectra and whether the radioactive isotope needs to be identified. Predominantly, nuclear security-based applications are interested in detecting either gamma-rays (typically E > 10 keV), and/or neutrons [6,7,8]. Gamma-rays are typically emitted from an excited nucleus going from a higher energy state to a lower energy state, usually following the decay of its parent nucleus. Several mechanisms, such as fission and fusion reactions, neutron capture reactions, annihilation reactions and activation processes, can all result in the emission of gamma-rays. Because gamma-ray assay and spectra measurements are the easiest and most common technologies, they are of tensed to identify and differentiate different nuclear materials and their isotopic composition [7]. Figure 1 shows the gamma-ray intensity spectra and characteristic peaks for various nuclear material isotopes [7,9,10].

**Figure 2:**
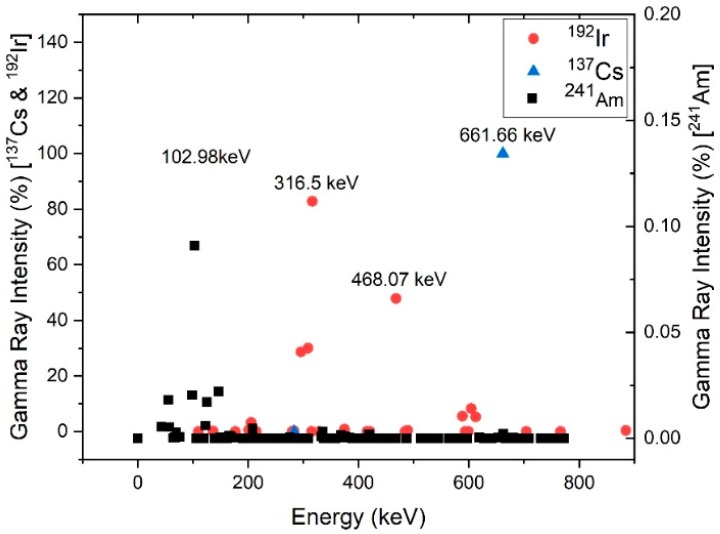
Gamma-ray characteristic energies and energy peaks of ^192^Ir, ^137^Cs and ^241^Am.

Other gamma emitting radiation sources that are often found to be involved in illicit trafficking are ^192^Ir, ^137^Cs and ^241^Am [2]. Figure 2 shows gamma-ray characteristic peaks of these three isotopes.

Neutron emission detection and neutron assay is another common procedure used to detect and identify nuclear materials and radiation sources [6,7]. Neutron sources in nature and industry can be categorised as spontaneous fission sources, reactor sources, alpha-neutron sources, photo-neutron (or gamma-neutron) sources and ion accelerator sources as shown in Table 1 [6,11,12,13].

Production of tritium from accelerator-based sources is affected by the closure of tritium-production reactors, non-proliferation policies and funding cuts. Other sources of tritium are breeding redactions in lithium blankets [14]. Other possible sources of neutron are D_Li-reactions [15] and spallation reactions [16]. Neutron multiplicity $\tilde{\upsilon }$, or the number of neutrons emitted per fission, is a parameter obtained in the result of an analysis or measure. Table 2 gives a list of spontaneous fission isotopes commonly subjected to neutron multiplicity assays [6,7,10,17].

**Figure 3:**
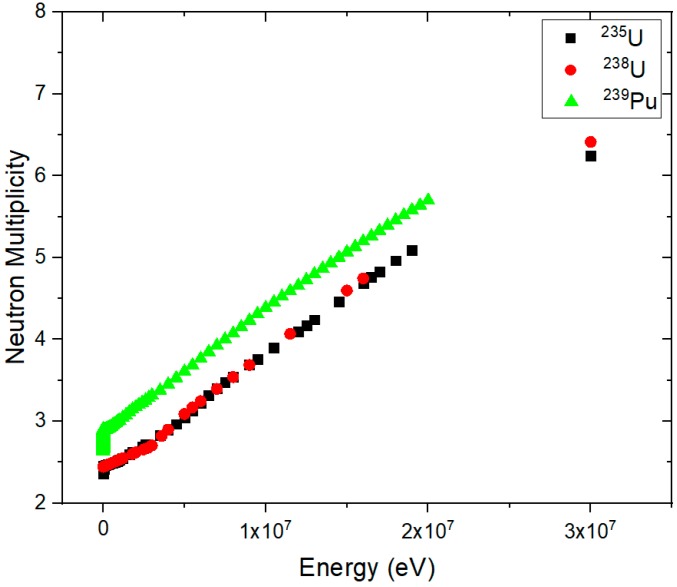
Neutron multiplicity as function of incident neutron energy for ^235^U and ^239^Pu.

Induced fission multiplicity depends on the fission isotopes and the energy of the incident neutrons [17,18]. Figure 3 illustrates neutron spectrum multiplicity for nuclear materials ^235^U and ^239^Pu as functions of energy.

Unlike gamma-rays, the wide energy spectrum of emitted neutrons, and the change in their energy as they traverse materials, make source identification through the energy of emitted neutrons a less effective method of detection. However, the increasing volume of research in this field such as the research in the field of neutron scattering cameras may indicate the emergence of new technologies [19,20,21,22,23].

## 3. Problem Definition and Authorities’ Requirements

The major concern involving illicit trafficking and proliferation of nuclear materials is the threat of using these materials in criminal activities and terrorist acts. This concern has been gradually increasing during the last three decades and is becoming a definite threat in times of international instability and travel. The subject of illegal nuclear trafficking and unlawful nuclear acts is becoming the primary concern of international and global agencies such as the IAEA [24], European Commission [25] and Interpol [26]. Other factors including the economic and political impacts of this illicit trafficking are also part of the multithreaded problem.

As with many illegal acts at the international level, security plans and prevention policies along with international legislation have been implemented to deter and prevent illicit trafficking and to promote nuclear non-proliferation. Examples of these plans and treaties are the Treaty on Non-proliferation of Nuclear Weapons in 1970 [27] and IAEA safeguards agreement and Code of Conduct on the Safety and Security of Radioactive Sources in 2004 [28]. Another example of international cooperation to deter illicit trafficking of nuclear materials is demonstrated by the adoption of the practices espoused in the Handbook of Nuclear Law produced by the IAEA [29]. This is the result of international organisations assisting legislation and regulatory bodies in member states in creating a strong and robust regulatory framework [29]. Other international, regional and cross continent agreements such as the International Convention for the Suppression of Acts of Nuclear Terrorism (ICSANT) [30] are part of the global effort to combat and prevent illicit trafficking of nuclear and radiological materials.

The safeguarding of radioactive materials in general is a continuous process, from the generation stage to the decommissioning stage, especially for nuclear materials. The uninterrupted tracking of these materials is the optimal method to safeguard and diminish the possibilities of illegal trafficking. While the situation norm is the controlled and legal transport of radioactive materials, incidents are still reported [2]. A series of protocols and procedures have been implemented at the national and international level to prevent these incidents. One of the most important factors in this process is the implementation of the means of detecting, identifying and localising radioactive materials using radiation detection equipment and radiation imaging techniques.

The main purpose of implementing detection and imaging technologies in these applications is the timely and accurate identification of illegal acts and the generation of evidence to enforce legal proceedings to eliminate trafficking networks [24,31]. The implementation of radiation detection and imaging technologies varies from state to state, but these technologies are generally implemented on sites where radioactive sources’ life cycles are spent, such as nuclear reactors, hospitals, etc., and at national and international cross borders. Many parameters affect the efficacy of radioactive material detection, with the main factor being the performance of the technologies employed, especially their ability to identify and localise radioactive sources [32]. Other directly related parameters that can influence the choice of technology employed are the field of view, the potential targets and the time constraints. The area of interest is the location where the detection or imaging instrument will be stationed and the zone that needs to be monitored. As implied in the Introduction, this area can be categorised as controlled or uncontrolled and varies in terms of the size of the area to be scanned, the detector to source distance, the number of people/vehicles/items to be monitored and the extent of the shielding or obstructions in the vicinity. The nature of the potential targets affects the choice of detection or imaging system due to their inherent shielding characteristics, i.e., nuclear material hidden inside the engine block of a large truck will be difficult to detect from a distance due to the significant shielding this environment affords. In addition, regulations that preclude the use of active interrogation systems on targets for health and safety reasons may also affect the selection process, if scanning pedestrians or queues of passengers, for instance. Timing is another parameter that affects the selection process. Controlled areas such as airports and land ports are busy areas. For example, the daily average number of people at a busy airport like Heathrow Airport is over 200,000 passengers per day [33]. There will be a limit to how long passengers can be held for security checks for logistical reasons. Therefore, detection efficiency, data analysis speed and spatial resolution are key aspects of the specification of the technologies employed. The size of the detection or imaging system can as well be seen as a factor on the selection process. Pocket-type instruments are used to detect the presence of radioactive materials and in some cases the radiation level, usually to calculate personal dose. Hand-held instruments have higher sensitivity and can be used to detect, locate and characterise radioactive sources. Finally, fixed and vehicle-based devices are usually used at borders cross-points, seaports and similar controlled areas [32].

The IAEA suggests that there are over a hundred different forms of non-destructive analysis techniques available to be used in the process of identifying radioactive materials [31]. However, the most common detection and imaging devices utilise gamma-rays and/or neutrons. The specification of suitable gamma-ray and neutron detection equipment varies according to legislation and the safeguarding abilities of states. A set of criteria have been recommended by the IAEA in a collaboration with World Custom Organization (WCO), EUROPOL and INTERPOL. The main components in this set of recommendations are [31,32]:

Gamma-ray systems’ requirements:

- At a mean dose rate of 0.2 μSv/h, the alarm of the system should be activated when the dose rate increases in a period of 1 s by 0.1 μSv/h for a pocket size instrument, by 0.05 μSv/h for a handheld instrument and 0.1 μSv/h for a fixed-installation instrument, for a duration of one second with 99% detection accuracy.
- False alarm rate should be minimal, with background measures of 0.2 μSv/h, with a false alarm rate of less than one every 12 h for pocket size instruments, less than six per hour for handheld instruments and less than one per day for fixed-installation instruments.

Neutron systems requirements:

- The alarm of the system should be activated above a threshold of 20,000 n/s with a source to detector distance of 0.25 m for handheld instruments and 20,000 n/s in 5 s with source to detector distance of 2.0 m for fixed-installation instruments, using a system with 99% detection accuracy.
- False alarm rate should be minimal with less than six per hour for handheld instruments and one per day for fixed-installation instruments.

Similarly, the American National Standard for Evaluation and Performance of Radiation Detection Portal Monitors for Use in Homeland Security have a set of criteria for gamma-ray and neutron equipment; however, the set of requirements are relative to initial reference settings within the equipment [34]. Applying these requirements might limit direct implementation and might affect the response of the system. Test and Evaluation Capabilities and Methodologies Integrated Process Team (TECMIPT) Test Operations Procedures (TTOP) For Radiation Detection Systems—Specific Methods specifies the minimum performance requirements for gamma-ray and neutron detection instruments [35]. These specifications have direct implementation and offer detailed requirements relative to the size category of the system.

Gamma-ray systems’ requirements:

- The alarm of the system should be activated when the count increases above the background level by 0.5 μSv/h in 2 s for Radionuclide Identification Devices (RIDs) in the pocket and handheld size categories.
- The alarm of the system should be activated with ^232^Th, ^137^Cs, and ^133^Ba, ^60^Co and ^57^Co sources moving past the system at a speed of 2.22 m/s and distance of closest approach of 3 m for RIDs in the fixed installation size category.
- False alarm rate should be minimal with less than one every 10 h for pocket size and handheld instruments and less than one every two hours for fixed-installation size instruments.

Neutron systems requirements:

- The alarm of the system should be activated when the exposure is above the threshold of 20,000 n/s in 2 s with ^252^Cf sources with a source to detector distance of 0.25 m for RIDs in the pocket size and handheld size categories.
- For a moving ^252^Cf source with activity of 20,000 n/s and moving past the system at a speed of 2.22 m/s at a distance of closest approach of 3 m, the system has to be able to detect the source with up to 1 cm steel or 0.5 cm of lead of shielding for RIDs in the fixed installation size category.
- False alarm rate should be minimal with less than one every 10 h for pocket size and handheld instruments and less than one every two hours for fixed-installation size instruments.

## 4. Physical and Electronic Collimations

Neutrons and gamma-rays are uncharged high-energy radiation fields. Conventional converging and diverging techniques, as well as other optical techniques, are not applicable in this case. A device is needed to precisely identify the lines along which detected radiation fields are generated. Collimation is the key word here. Collimation of incident radiation can be done physically and/or electronically. Physical collimation and electronic collimation are well-established imaging techniques in the field of radiation detection. The basic concepts of each of these two collimation technique are discussed in this section.

### 4.1. Physical Collimation

Physical collimators are patterns of highly attenuating materials positioned in front of a detector to limit the direction of incident radiation quanta to specific directions. As a result, a shadow image is formed on the detector resulting in greatly improved spatial resolution. However, this approach causes a noticeable decline in the efficiency of the system since it limits the number of detectable radiation quanta [36]. Physical collimation for gamma-rays is more effective at lower energies as the probability of penetration through matter increases with gamma-ray energies above the energy peak of Compton scattering.

**Figure 4:**
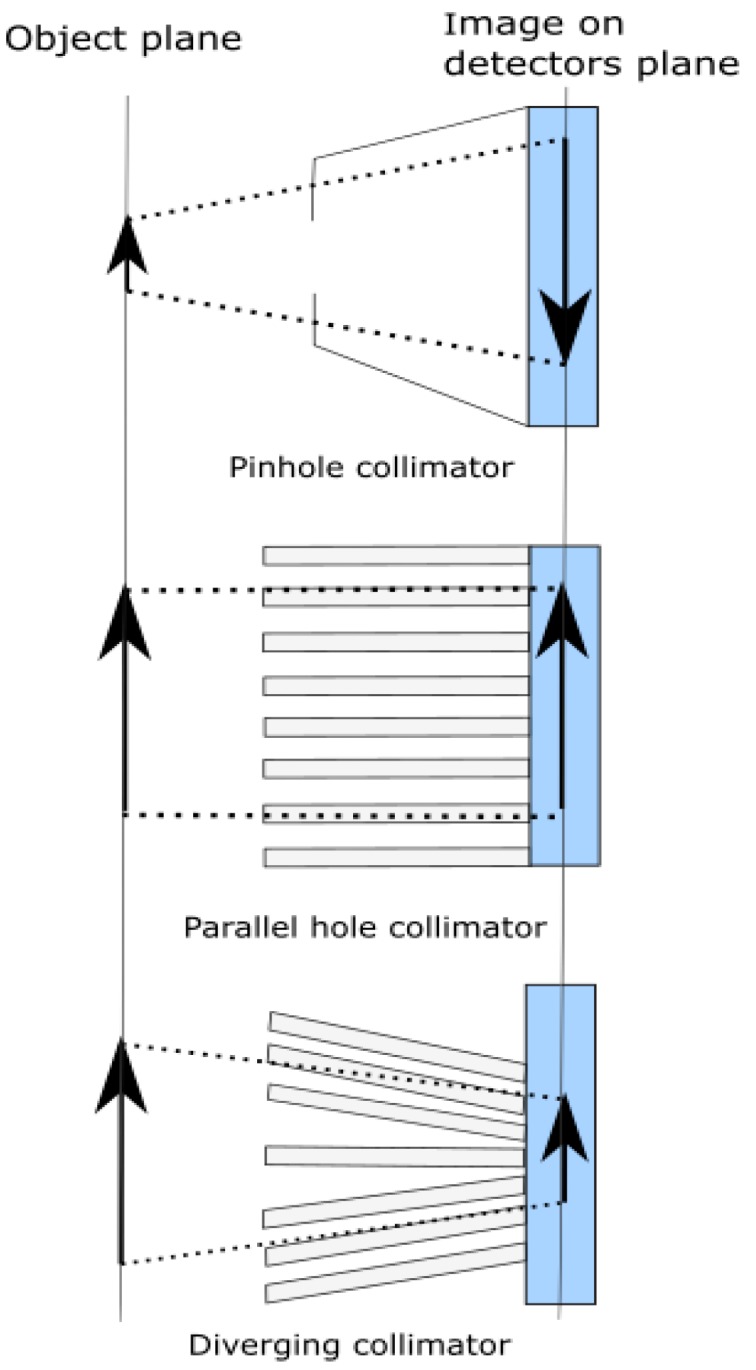
Schematic of physical collimator types.

The simplest physical collimator design is the pinhole collimator, which consists of a single small aperture. This technique offers excellent angular resolution; however, it limits the geometrical efficiency of the system. Parallel holes collimator, converging and diverging collimators are arrays of opaque and transparent photon channels used in imaging where the system scans across the entire field of view. The technique improves the angular resolution of the system and slightly increases the solid angle. Figure 4 shows schematics of physical collimators types.

**Figure 5:**
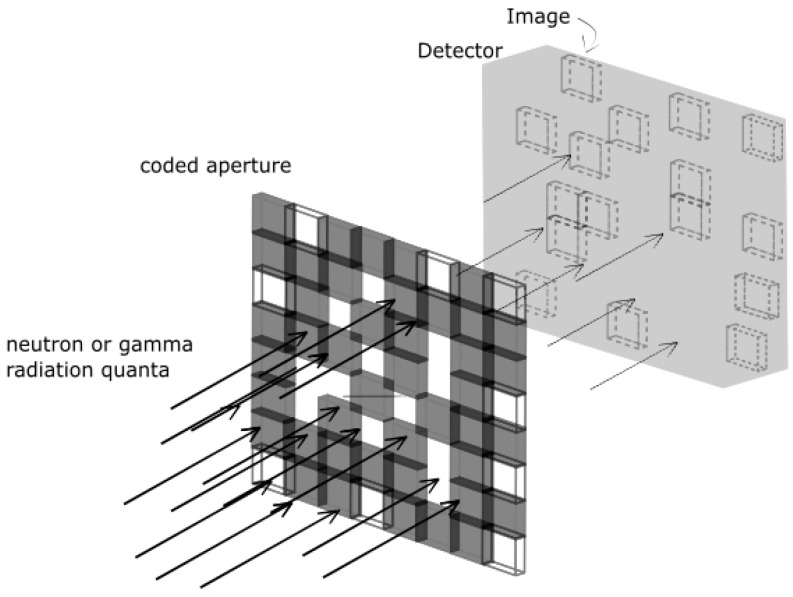
A schematic of coded aperture imaging system, with a coded aperture mask that is made with a pattern of opaque and open cells of highly attenuating materials followed by a radiation sensitive detector. Incident radiation field is attenuated in the coded aperture mask, with only a fraction of incident radiation is transmitted and detected on the system (based on reference [37]).

A coded aperture is an alternative and popular form of physical collimation that was originally proposed for astrophysics measurements. It was first analytically proven effective for imaging systems in 1968 [37,38]. Commonly based on a 50% open mask with a large number of randomly distributed pinholes lying in a parallel plane with the detector, the technique offers higher efficiency compared to previously mentioned collimation techniques. Figure 5 illustrates the basic parameters of coded aperture imaging systems.

Coded aperture masks have greatly evolved since their inception, and, in most cases, their design can be tailored to fit the application requirements. There are generally two types of coded apertures: passive masks and active masks. In the case of passive masks, a highly absorbing material is used to stop and eliminate non-normally directed radiation quanta from reaching the detector. The choice of materials in passive masks mainly depends on the type of target radiation. High density/atomic number materials such as lead, tungsten and depleted uranium are often used to block high energy photons, while neutron absorbing materials such as high-density polyethylene (HDPE) and Gadolinium are used in coded apertures for neutron detection [39,40]. Passive physical collimation shows noticeable drawbacks over a considerable range of the energy spectrum, especially at high energies where radiation fields have enough energy to penetrate the opaque pattern of the mask [41,42,43]. On the other hand, active coded aperture designs use radiation sensitive materials, such as B- and Gd-doped glass plates for detecting low energy neutrons, as part of the collimation and detection process, which allows the detection of radiation quanta with a wider energy range [44,45,46,47]. Most of these active collimation examples combine physical collimation and Compton scattering in one system by using a pattern of large area detectors. Physical collimation is mainly utilised for detection of low gamma-ray energies, while Compton scattering is utilised for higher energy gamma-rays. Generally, the trade-off between angular resolution and detection efficiency is unavoidable in physical collimation. Higher activity sources or longer acquiring times (or both) are usually recommended to improve the efficiency of these systems.

### 4.2. Electronic Collimation: Compton Camera and Neutron Scattering Camera

**Figure 6:**
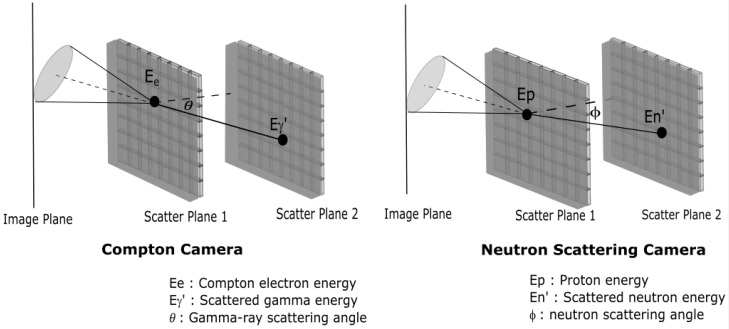
Schematic of basic parameters in Compton scattering camera (**left**) and neutron scattering camera (**right**).

Electronic collimation (widely known as a Compton camera for gamma-ray detection and neutron scattering camera for fast neutron detection) is a well-studied collimation approach, especially utilised within gamma-ray detection. Gamma Compton cameras are comprised of two pixelated detectors and utilise the laws of conservation of momentum and energy to infer the most probable trajectories of the scattered and/or absorbed radiation fields. The first detector scatters the gamma photon, which results in an electron being emitted and its energy measured. The second detector absorbs the scattered gamma photon and measures its energy. The location of the pixels activated in each detector determines the angle of scattering and hence the probable origin; the energy of the initial gamma photon can be calculated from measured energies of the incident and scattered photons [42,48]. The neutron scattering camera similarly utilises at least two detectors and the conservation of energy and momentum. However, in this instance, the reaction is between an incident fast neutron and a proton present in the proton-rich detectors in order to sense and localise the fast neutron source. The time-of-flight data of the scattered neutron is used to measure the energy of the incident neutron [19,23]. Figure 6 shows the basic elements in a two pixelated planes imaging system based on electronic collimation.

## 5. Passive Detection Systems of Illicit Radioactive Materials

The two modes of detecting nuclear materials and other radioactive sources are mainly active mode and passive mode. Active detection mode (not part of the work presented here) uses externally generated neutrons, gamma-rays or X-rays to interrogate radioactive materials. This approach offers in-depth characterisation of target radioactive material, especially for fissile materials, although the major drawbacks are that it cannot be used in many circumstances, such as in proximity with humans and in uncontrolled detection areas [6,49,50,51,52].

In passive detection, an imaging system is used to detect and characterise neutrons and gamma-rays directly emitted from nuclear materials and radioactive sources. In contrast to an active detection technique, passive detection requires less architecture arrangement and conceivably lower in cost. In Safeguards Techniques and Equipment series by IAEA, approximately all gamma-ray non-destructive equipment discussed in the report are in passive mode [31]. In the same report, the ratio of listed passive to active neutron assay equipment is 4:1. This clearly shows the impact of passive detection mode at the international level in safeguard and security applications. A common design is the Radiation Portal Monitor (RPM), which typically consists of several detectors designed in a rectangle shape located at a fixed site [5]. Some passive imaging systems can characterise the radioactive material, reject background radiation and estimate the source to system distance. Passive detection systems offer a safe and simple detection mode, although the drawback is that its absolute efficiency decreases with increasing shielding around the radioactive material [53]. Since passive detection depends exclusively on the radioactive source under investigation and the detection system used, the statistical quality of results and the time to detect a source of specified strength depends mainly on characteristics such as intrinsic efficiency, angular resolution, spatial resolution and time resolution, shielding, and source-detector distance.

In this work, passive detection and identification systems are categorised based on the target radiation field: gamma-rays, neutrons and dual systems. In each category, the systems will be further classified into pocket-type instruments, hand-held instruments and large fixed or vehicle based instruments [54]. Another equally important classification factor is the purpose of detection instruments summarised as detection, assessment and localisation, and identification [54]. The following review attempts to compare and appraise past and present passive detection systems and techniques found in the literature that have been predominantly designed to detect and deter illicit trafficking, smuggling and transporting of nuclear materials and other radioactive sources.

### 5.1. Gamma-Ray Detection Systems

Common single crystal inorganic gamma detectors such as NaI, CsI, SrI2(Eu) and PVT (polyvinyltoluence organic scintillation detectors) or CdZnTe and HPGe (High Purity Germanium semiconductor detectors) are popular due to their stable performance, high efficiency and relatively low price [31,55,56,57,58]. NaI(Tl) is by far the most studied and most commercially successful inorganic scintillator [8]. However, single crystal imaging systems are far more sensitive to background radiation and are more prone to false alarms [3,59]. Pairing single crystal detector with signal analysers, such as multichannel analysers, might widen the scope of applications for this group of detectors [31]. However, imaging is almost always desirable, alongside detection, to enhance a system’s sensitivity, angular resolution, energy resolution and localisation of point-like sources [60,61].

Physical collimation, in particular coded apertures, and Compton scattering techniques have both been adopted to enhance and improve the detection abilities of gamma imaging systems. Fixed installation coded aperture systems offer long distance and large area coverage with improved signal-to-background ratio [62,63]. However, these systems are best implemented at border controls, as they require fixed or slowly moving targets. Problems and limitations, such as false alarms and timing issues, as well as proposed solutions for this technology, such as energy windows and baseline suppression, are frequently discussed in literature [64,65,66,67,68,69,70]. Hand-held coded aperture systems offer a flexible solution for detecting and localising of radioactive materials [71,72]. In addition to the main goal of detecting and localising radioactive sources while scanning vehicles, people, luggage and cargo, other applications such as monitoring the extent of nuclear related emergencies have been suggested. Many mechanically collimated systems have found success in this field [72,73,74,75]. Table 3 summarises coded aperture-based gamma imaging systems found in the literature, including their detection method, their size category and the purpose of application.

In the energy range of nuclear material gamma-ray sources (60 keV to 3.0 MeV), Compton scattering is the dominant photon interaction mechanism, which makes the Compton scattering technique the most appropriate technique compared to other techniques [3]. Compton based systems feature a wide field of view with improved detection efficiency compared to mechanically collimated gamma imaging systems, especially for high-energy gamma-rays [3,80]. In addition, Compton systems offer the ability to detect, assess and localise a gamma-ray source with an associated reduction in background radiation [81]. Fixed installation and portable Compton systems are the most common size categories [82,83,84,85]. The performance of these systems varies between detectors with some using low energy resolution, high sensitivity NaI(Tl) and CsI(Tl) scintillations [82,83], while others use high resolution Si and HPGe semiconductor detectors [84]. Image reconstruction methods for Compton systems, such as Maximum Likelihood Expectation Maximization (MLEM), Maximum Likelihood Ratio (MLR) and stochastic origin ensembles, have been regularly studied and optimised for their direct impact on the performance of Compton system in this field [86,87]. There are hybrid-imaging systems that utilise both Compton camera and coded aperture technology; examples include passive mask [88] and active mask [45,89,90] systems. The duality in imaging techniques aims to utilise the advantages of both physical collimation and Compton scattering. However, designs need to take into account the optimum arrangement of layers to avoid negating these advantages. Another promising technique in gamma-ray imaging are 3D systems that utilise coded apertures or Compton scattering. The 3D systems are used in assessing and localising smuggled and hidden sources by projecting a 3D image of the search scene, which allow faster and easier navigation in the area of interest [91,92].

### 5.2. Neutron Detection Systems

Although most nuclear materials emit either or both neutron and gamma-rays, heavy shielding of gamma-rays can greatly lower the efficiency of gamma-ray imaging systems, negatively impacting their efficacy in nuclear materials’ non-proliferation and safeguard applications. Neutrons are highly penetrating and nuclear materials emitting neutrons require bulky shielding to completely conceal neutrons. Therefore, neutron imaging systems are extensively used in nuclear materials imaging and they offer an excellent alternative. Due to their high thermal neutron cross section (5330 barns) and low gamma-ray sensitivity, ^3^He gas filled counters have been the standard neutron monitoring technology for decades [31,93]. Thermal neutrons detection efficiency for ^3^He gas filled counters is a function of the amount of ^3^He gas and increases with increasing pressure. For example, a 72 in in height and 2 in in diameter ^3^He tube under 3 atm pressure has efficiency of 3.05 cps/ng ^252^Cf. The main supply of ^3^He is the ^3^H purification process, which has seen a dramatic decrease in the last two decades [93,94]. This has led to a continuous search for alternative neutron detection technologies. Direct gas filled counter alternatives such as BF_3_ proportional counters, boron lined proportional counters and fission chambers have been commercially in use [31,95,96,97], but they have been shown to be significantly less efficient [94,97,98].

Neutron sensitive scintillation detectors and semiconductor detectors are frequently used in neutron detection. Neutron sensitive scintillation detectors include liquid and plastic organic scintillators [99,100,101,102,103,104], glass scintillators [105,106,107,108] scintillating fibres [109] and bubble chambers [110]. Bonner spheres are examples of radiation detectors embedded in a spherical moderator layer. Bonner spheres are well-established neutron spectrometer instruments in the field of nuclear dosimetry and inspection non-proliferation [111,112]. However, Bonner spheres have inherently low energy resolution and inverse relationship between moderator thickness and detection efficiency. Semiconductor based detectors are a less popular means of neutron detection due to their lower efficiency compared to the scintillation detection materials in this field and the occasional requirement of having to use foils or coatings of conversion material to convert neutrons into a detectable signal, usually electrons [13,113]. However, their ruggedness and high-speed response make them an interesting option for safeguarding and security applications [114,115]. Semiconductor materials such as ^4^H-SiC, diamond and CdZnTe have been investigated in literature for their applications in neutron detection [116,117,118,119]. ^4^H-SiC and SiC semiconductors are promoted for their abilities to work in high temperature and high radiation environments along with other desirable properties such as high energy band gap and lower production cost, compared to diamond, which has similar properties [116]. Diamond materials, such as diamond high pressure, high temperature (HPHT) synthetic diamond or diamond grown using CVD, are mechanically durable and inherently radiation hard. Like SiC detectors, diamond detectors have a wide band gap, which makes them highly appealing for radiation detection applications at high temperatures [118,120]. CdZnTe with neutron converting layer such as Gd are proposed for portable thermal neutron detection systems [119,121]. Activation foils were suggested for safeguard applications such as practical neutron flux measurement tools [122].

As for gamma-ray detection, collimation techniques in neutron detection are deployed to enhance detection efficiency, angular and energy resolutions, increase the field of view and decrease the acquisition time. In addition, for screening vehicles, cargo and large containers, the imaging systems should be accurate with a low probability of false alarms and low sensitivity to gamma-rays [123]. A number of simulation-based studies discuss potential neutron imaging systems with physical collimation or neutron scattering/ToF (Time of Flight) based collimation [124,125,126,127]. An equally important aspect in nuclear materials detection is the discrimination method used to discriminate between neutrons and gamma-rays [128]. Because gamma-rays are almost always present in the background, discrimination methods are crucially important and have been extensively studied in literature [129,130,131,132,133,134]. A range of radiation detection and identification systems are commercially available from vehicle size [135] to handheld size [136,137,138]. For a more detailed review of portal radiation monitors, Table 4 lists all neutron-imaging systems discussed and experimentally evaluated in literature for safeguard and non-proliferation of nuclear materials. The categorisation of proposed applications and the size definitions are based on those mentioned in Section 4.

### 5.3. Dual Gamma-Ray and Neutron Detection Systems

Dual particle imaging systems detect gamma-rays and neutrons simultaneously and can differentiate between the two radiations. This method of imaging has an advantage over single particle imaging methods because it allows the passive detection and identification of a wide range of nuclear materials and other radioactive sources.

There are two main groups of systems in the field of dual particle imaging. The first group is comprised of single materials that are sensitive to both gamma-rays and neutrons. The second group uses multiple detection materials systems with detectors not necessarily sensitive to both particles. The latter imaging technique offers a reduction in system complexity as additional discrimination techniques are not necessarily required. In addition, this category offers higher design flexibility, as the parameters employed to enhance system response to one radiation field are usually independent of the other.

Materials sensitive to both gamma-ray and neutron have been investigated for their dual detection abilities since the 1950s [159,160]. Examples of the list of detection materials range from inorganic scintillators [161,162,163], semiconductor detectors [164,165,166], glass organic scintillators [167], some classes of elpasolite scintillators [168,169], some classes of liquid scintillators and plastic scintillators [170,171,172,173,174]. The common feature between these detection materials is their superior ability to enable the distinguishing of gamma-ray signals from neutron signals by methods such as pulse shape discrimination and pulse height discrimination [133,134,170,175]. A handful of fixed installation and portable monitoring systems are suggested for security and non-proliferation applications are found in the literature. The scintillation materials used in these systems vary dramatically with ^6^Li(Eu) and Li-glass detectors, EJ-309 liquid scintillators and CLYC (Cs_2_LiYCl_6_:Ce) elpasolite detectors being popular [162,176,177,178] with some also utilising coded aperture collimation to enhance imaging characteristics [179,180]. A number of examples of hand-held and pocket size systems for monitoring purposes similarly exist; almost all detection materials in this size category are based on plastic scintillators or elpasolite scintillation materials [99,181,182,183,184]. These systems offer flexibility and fast response, albeit with a limited field of view.

Since 2004, the research on multiple detection materials imaging systems for security and non-proliferation applications has increased. System abilities vary according to the detection and collimation method and the size of the system. Table 5 presents a timeline of multiple detectors imaging systems discussed in literature along with their collimation and detection techniques between 2004 and 2016.

A number of research papers have been undertaken and investments have been made into large area coverage using a network of detectors. This concept has been around for over a decade [194,195,196]; however, the realisation of the advantages of this technique along with the advances in network and communication fields will lead to new developments in this area. Examples of network systems and algorithms in this field are the RAdTrac network system for gamma detectors, the particle filter algorithm for a network of gamma counters and the ROSD-RSD (Ratio of Squared Distance-Radiation Source Distance) algorithm method [197,198,199,200]. Other systems like identiFINDER S900 [201] and SmartShieldTM v2.0 [202] are commercially available for radionuclides identification and tracking.

## 6. Conclusions

Illicit trafficking of nuclear materials and radioactive materials is a cross-border problem that must be tackled globally. Robust and efficient detection equipment and radiation detection systems stand on the front line of defence against the acts of illicit trafficking. However, understanding the different parameters that affect the choice of detection equipment and/or radiation detection systems can greatly help with installing the most effective detection techniques. The parameters that have the most effect are (more details in Section 3):

- Security agencies and legislation bodies requirements,
- Areas under surveillance and place of implementation,
- Image quality requirement,
- Timing and speed requirements.

Once the main requirements are established, the options can then be investigated within detection and/or imaging techniques of gamma-ray sensitive systems, neutron sensitive systems or dual gamma-ray and neutron sensitive systems. Each technique has its advantages over the others and the main stage in planning to install a detection system that will positively contribute in deterring illicit trafficking is to investigate and study each implementation site individually.

## Figures and Tables

**Table 1:** Neutron sources and average energies.

Neutron Source	Neutron Source Type	Average Neutron Energy (MeV)	Half-Life (Years)
^252^Cf	Spontaneous fission	1–3 (2.35 ^1^)	2.645
^241^Am-9Be	Alpha-neutron source	4.2	432.2
^239^Pu-9Be	Alpha-neutron source	4–5	24,114 years
^124^Sb-9Be	Photo-neutron source	0.025 (close to mono-energetic)	0.164 (60 days)
D-D reaction	Accelerator source	2.4 (close to mono-energetic)	N/A
D-T reaction	Accelerator source	14.1(close to mono-energetic)	12.32

^1^: Reference [8], page 93.

**Table 2:** Spontaneous fission isotopes and neutron multiplicity.

Isotope	Neutron Number	Total Half-Life (years)	Average Spontaneous Fission Multiplicity
^242^Cm	146	0.447	2.528
^249^Bk	152	0.877	3.4
^252^Cf	154	2.645	3.768
^248^Cm	148	3.84	3.161
^240^Pu	146	6.56	2.151
^238^Pu	144	87.7	2.21
^238^U	143	4.47 × 10^9^	2.0
^235^U	146	7.04 × 10^8^	1.87

**Table 3:** Coded aperture-based gamma-imaging systems.

System Size Definition	Examples and Proposed Application in Literature	Detector/s	Industrial Designation
Fixed installation	Detection and localisation [62,63]	CsI(Na)	
Fixed installation	Detection, assessment and localisation [76]	HPGe & NaI	MISTI
Fixed installation	Detection, assessment and localisation [75]	CdZnTe	ORIGAMIX
Fixed installation/hand-held	Detection and localisation [77]	NaI	RMC
Fixed installation/hand-held	Detection and localisation [73]	CsI(Tl)	CARTOGAM
Fixed installation	Detection, assessment and localisation [78]	(GSO)	
Hand-held	Detection and localisation [71]	CdTe-Medpixi2	
hand-held	Detection and localisation [74]	CsI(Na)	RADCAM
hand-held	Detection and localisation [79]	CsI(Tl)	
hand-held	Detection and localisation [72]	CdZnTe-Timepix	GAMPIX

**Table 4:** Examples of neutron imaging systems used in nuclear materials security, their collimation technique, properties and characteristics.

Author, Year and Reference	Proposed Application	Collimation/Detection Technique	System Size Definition	Main Detection Materials	Approximate Intrinsic Efficiency (Thermal Neutrons/Fast Neutron 252Cf) (%)
Miller et al. (2003) [139]	Detection, assessment and localisation	Neutron scatter	Fixed installation	Plastic scintillator	NA/NA
Bravar et al. (2006) [140]	Detection and assessment	Neutron scatter	Fixed installation	BC-404 plastic scintillator	NA/NA
Vanier et al. (2007) [141]	Detection, assessment and localisation	Neutron scatter	Fixed installation	Plastic scintillator	NA/NA
Mascarenhas et al. (2009) [142]	Detection, assessment and localisation	Neutron scatter	Fixed installation	EJ-301	NA/NA
Siegmund et al. (2009) [143]	Detection	Coded aperture and Stack of microchannel plates	Fixed installation	^10^B doped microchannel plates	~20%/NA
Herbach et al. (2010) [144]	Detection and assessment	Null/gamma from neutron capture	Fixed installation	BGO with Cd converter	45%/NA
Ryzhikov et al. (2010) [145]	Detection and assessment	Null/gamma from neutron capture	Fixed installation	CdWO_2_	67%/42%
Marleau et al. (2010) [146]	Detection and assessment	Active coded aperture	Fixed installation	EJ-301	NA/NA
Nakae et al. (2011) [147]	Detection and assessment	Null/Array of liquid scintillator	Fixed installation	Organic liquid scintillator (not specified)	NA/~6% (absolute)
Bellinger et al. (2012) [148]	Detection and assessment	Null/array of slabs	Hand-held	Si diodes with ^6^LiF	6.8%/NA
Ide et al. (2012) [149]	Detection, assessment and localisation	Neutron scatter	Fixed scintillator	EJ-309	NA/NA
Joyce et al. (2014) [150]	Detection and assessment	Null/Multiplicity assay	Fixed scintillator	EJ-309	NA/
Brennan et al. (2015) [151]	Detection, assessment and localisation	Coded aperture and Time-encoded imaging	Fixed installation	Organic liquid scintillator (not specified)	NA/NA
Fronk et al. (2015) [152]	Detection and assessment	Null/double sided Microstructure	Hand-held	Si diodes with ^6^LiF	~29.48%/NA
Ianakiev et al. (2015) [153]	Detection and assessment	Null/^6^Li embedded in PVT	Fixed installation	^6^Li and PVT	NA/NA
Hoshor et al. (2015) [154]	Detection and assessment	Null/array of slabs	Hand-held	Si diodes with ^6^LiF	~22%/~4.5%
Goldsmith et al. (2016) [155]	Detection, assessment and localisation	Neutron scatter	Fixed installation	EJ-309	NA/45%
Fulvio et al. (2017) [156]	Detection and assessment	Ring of multiplicity counters	Fixed installation	EJ-309	NA/
Cowles et al. (2018) [157]	Detection and assessment	Null/multiple panels	Fixed installation	LiF/ZnS	36%/NA
Ochs et al. (2019) [158]	Detection	Microstructure semiconductor	Wearable device	Si diode with ^6^LiF	30%/NA

**Table 5:** Timeline of dual particle multiple detectors imaging system in security and non-proliferation applications.

Year	Author and Reference	Collimation	Main Detection Materials
2004	Aryaeinejad and Spencer [185]	None	^6^Li and ^7^Li-loaded glass scintillators
2007	Baker et al. [186]	None	NaI(Tl) and LiI(Eu)
2008	Enqvist et al. [187]	None	Cross correlation BC-501A
2009	Runkle et al. [188]	None	NaI(Tl) and ^3^He
2011	Polack et al. [189]	Compton and neutron scattering	NaI(Tl) and EJ-309
2012	Cester et al. [190]	None	LaBr(Ce), NaI(Tl), NE-213 and ^3^He
2013	Ayaz-Maierhafer et al. [191]	Coded aperture	CsI and EJ-309
2014	Poitrasson-Rivière et al. [192]	Compton and neutron scattering	NaI(Tl) and EJ-309
2016	Cester et al. [193]	Null	EJ-420, EJ-560 and EJ-299-33A
